# Editorial: Neutrophil Functions in Host Immunity, Inflammation and Tissue Repair

**DOI:** 10.3389/fimmu.2021.810346

**Published:** 2021-11-30

**Authors:** Sonja Vermeren, Philip M. Elks, Felix Ellett

**Affiliations:** ^1^ Centre for Inflammation Research, Institute for Regeneration and Repair, The University of Edinburgh, Edinburgh, United Kingdom; ^2^ Department of Infection, Immunity and Cardiovascular Sciences, The University of Sheffield, Sheffield, United Kingdom; ^3^ Center for Engineering in Medicine and Surgery, Massachusetts General Hospital, Harvard Medical School, Boston, MA, United States

**Keywords:** neutrophils, trafficking, migration, inflammation, immunity, repair

Neutrophils are the most abundant circulating leukocytes in humans and are amongst the first responders to be recruited to sites of injury and infection in response to inflammatory cues. These short-lived cells are formed by granulopoiesis in the bone marrow. Mature neutrophils are released into the circulation in a chemokine-regulated, circadian fashion (1). In the absence of stimulatory signals, neutrophils will circulate for ~1 day before becoming senescent and homing back to the bone marrow. In the presence of stimulating signals, neutrophils migrate to inflammatory sites along gradients of chemokines. Exit from blood vessels is usually selectin- and integrin-dependent, although certain sites with highly specialised vasculature (e.g., lung, liver, brain) are characterised by extravasation that is supported by alternative adhesion molecules (2). In contrast, interstitial neutrophil migration occurs in an integrin-independent fashion. Upon reaching inflammatory sites, neutrophils clear cell debris and act as the first line of cellular immunity against invading pathogens. Once their job is done, neutrophils either undergo apoptosis, releasing “eat-me” signals and are cleared by professional phagocytes; or can migrate away from inflamed tissues, promoting tissue healing responses. If these processes are dysregulated, neutrophils can release their toxic components into surrounding tissues, leading to bystander cell damage and chronic inflammation.

This Research Topic on neutrophil biology aims to highlight some of the latest developments in the diverse roles of neutrophils in host immunity, inflammation and tissue repair.

Neutrophils become involved in inflammatory processes through rapid, directed migration towards sites of tissue damage. Bader et al. examined the β2 integrin-dependency of neutrophil recruitment to transected tailfins in zebrafish larvae *via* generation of a CRISPR/Cas9 genetic deletion of CD18, identifying a reduction in neutrophil trafficking to the sterile inflammation site, alongside an increase in neutrophil number found in the circulation. Two reports evaluated the contribution of individual signaling intermediates in diverse neutrophil functions, with a particular focus on neutrophil trafficking. Yan et al. explored the function of the Gαi2 – regulator of G (RGS) protein interactions, showing that they are required for fine-tuning neutrophil trafficking as well as clearance of aged neutrophils, ultimately avoiding excessive neutrophilic inflammation. Meanwhile, Michael et al. reported that the phosphoinositide 5-phosphatase SHIP2 promotes neutrophil trafficking to sites of inflammation and chemotactic directionality. Directed neutrophil migration is not limited to chemical cues, but can also occur in electric fields in a process called electrotaxis, a phenomenon investigated by Moarefian et al. using microfluidics, where they showed that neutrophil migration can be rapidly modulated by electric fields.

Neutrophils do not act alone, they also interact with multiple other cell types during their lifetime that help orchestrate the wider immune response (3). Richardson et al. contributed a review on devices that are suitable to investigate neutrophil cross-talk with other cell types during neutrophil migration. In the lymph node, neutrophils interact with T cells, either promoting or suppressing T cell proliferation. Minns et al. show how the nature and outcome of these interactions is dependent upon the activation status of both the T cell and the neutrophil. Neutrophils are also able to communicate with each other and can self-amplify external chemokine gradients, for example by producing the lipid chemoattractant LTB4, resulting in the formation of formidable neutrophil swarms (4). Emerging evidence suggests that neutrophil swarming involves other cell types. In their report, Walters et al. examined neutrophil-monocyte cross-talk in the context of swarming *in vitro*.

In addition to their well-characterised role in generating inflammation, neutrophils are also involved in promoting the resolution of inflammation and relatedly tissue repair (5). One important contribution towards the resolution of inflammation stems from neutrophils undergoing a non-inflammatory cell death, apoptosis, followed by efferocytosis (phagocytosis of apoptotic neutrophils) by macrophages and release of anti-inflammatory cytokines (6). Sekheri et al. contributed a review on the role of the versatile neutrophil β2 integrin Mac-1, focussing on its function in promoting resolution in contexts such as phagocytosis-induced cell death. Dobosz et al. analysed a molecular mechanism underpinning the induction of neutrophil apoptosis, reporting that monocyte induced chemoattractant protein-induced protein 1 (MCPIP-1) mediated degradation of anti-apoptotic transcripts, with MCPIP-1 transcript stability in turn subject to regulation by miRNAs. In contrast, Szeifert et al. reported that Mac-1 clustering promotes the release of pro-inflammatory, antibacterial extracellular vesicles from neutrophils. Two further reports analysed molecular signalling events, with Paré et al. examining molecular events involved in the dampening down of neutrophilic inflammation *via* the inhibitory C-type lectin receptor CLEC12A, while Alemán et al. characterised Fc receptor-induced, TRPM2 calcium channel-mediated entry of extracellular Ca^++^ into neutrophils.

The neutrophil processes described above are often dysregulated in disease, leading to neutrophil-driven tissue damage and chronic inflammation. A case in point is diabetes, in which the roles of neutrophils in driving disease is the subject of a review article by Dowey et al. A research paper on diabetes by Insuela et al. addressed how glucagon interferes with neutrophil chemotaxis, promoting susceptibility to sepsis, a common complication in diabetic patients. Chen et al. showed that neutrophil dysregulation in sepsis is subject to miRNA-dependent transcriptional regulation. Hayes et al. focussed on the role of poorly controlled phagosome pH in impaired intraphagosomal microbial killing in the context of cystic fibrosis. In addition to promoting bacterial infections, neutrophil dysregulation also contributes to sterile chronic inflammation. Van Nevel et al. identified neutrophilic inflammation in a model for allergic asthma in BALB/c but not C57BL/6 mice. How neutrophil dysregulation promotes rheumatoid arthritis and systemic lupus erythematosus is the subject of a review by Fresneda Alarcon et al. Abundant neutrophil extracellular traps (NETs) are one feature of these autoimmune diseases that are thought to be instrumental in disease progression, in addition to their important function in host immunity (7). A report by Tatsiy et al. examines the timing of critical molecular events that underpin NET formation.

The potential of neutrophil heterogeneity is an exciting and emergent discussion in the field (8). In particular, low-density neutrophils have been described in a range of disease states, and have been characterised by different sets of markers depending on the disease with which they are associated. McKenna et al. reviewed markers associated with these different neutrophil subsets. Neutrophil heterogeneity and low-density neutrophils were also the subject of several research articles in this topic with Bongers et al. examining subsets in disease states, while reports by Blanco-Camarillo et al. and by Hardisty et al. analysed low density neutrophils in healthy individuals, suggesting that these cells are not only associated with particular disease states but may be normally present during homeostasis and health.

We would like to thank all the contributors to this Research Topic and the reviewers for generously giving their time and expertise. We trust that this Research Topic on the roles of neutrophils in host immunity, inflammation and tissue repair gives the readers a sense of the cutting-edge research being done in this field.

## Author Contributions

All authors listed have made a substantial, direct and intellectual contribution to the work and approved it for publication

## Funding

SV is supported by the MRC (MR/S008020/1). PE is a Sir Henry Dale Fellowship jointly funded by the Wellcome Trust and the Royal Society (Grant Number 105570/Z/14/A). FE acknowledges support from the NIH (grant GM092804).

## Conflict of Interest

The authors declare that the research was conducted in the absence of any commercial or financial relationships that could be construed as a potential conflict of interest.

## Publisher’s Note

All claims expressed in this article are solely those of the authors and do not necessarily represent those of their affiliated organizations, or those of the publisher, the editors and the reviewers. Any product that may be evaluated in this article, or claim that may be made by its manufacturer, is not guaranteed or endorsed by the publisher.
